# A novel anatomic titanium mesh cage for reducing the subsidence rate after anterior cervical corpectomy: a finite element study

**DOI:** 10.1038/s41598-021-94787-0

**Published:** 2021-07-28

**Authors:** Yuhang Wang, Yi Zhan, Huiming Yang, Hua Guo, Haiping Zhang, Qinpeng Zhao, Dingjun Hao, Biao Wang

**Affiliations:** 1 Department of Spine Surgery, Honghui Hospital, Xi’an Jiaotong University College of Medicine, No. 76 Nanguo Road, Xi’an, 710054 Shaanxi China; 2 Xi’an Medical University, No. 74 Hanguang North Road, Xi’an, 710068 Shaanxi China; 3 Shaanxi University of Chinese Medicine, No.1, middle section of Shiji Avenue, Xianyang, 712046 Shaanxi China; 4 Department of Orthopaedics, Shehong Municipal Hospital of TCM, No. 239 Meifeng Avenue, Taihe Street, Shehong, 629200 Sichuan China; 5 Department of Orthopaedics, Xi’an Central Hospital, Xi’an Jiaotong University College of Medicine, No. 161 Xiwu Road, Xi’an, 710003 Shaanxi China

**Keywords:** Clinical trial design, Translational research

## Abstract

Fusion with a titanium mesh cage (TMC) has become popular as a conventional method after cervical anterior corpectomy, but postoperative TMC subsidence has often been reported in the literature. We designed a novel anatomic cervical TMC to reduce the postoperative subsidence rate. According to the test process specified in the American Society of Testing Materials (ASTM) F2267 standard, three-dimensional finite element analysis was used to compare the anti-subsidence characteristics of a traditional TMC (TTMC) and novel TMC (NTMC). Through analysis, the relative propensity values of a device to subside (Kp) of the TTMC and NTMC were 665.5 N/mm and 1007.2 N/mm, respectively. A higher Kp measurement is generally expected to indicate that the device is more resistant to subsidence into a vertebral body. The results showed that the novel anatomic titanium mesh cage (NTMC) significantly improved the anti-subsidence performance after anterior cervical corpectomy and fusion (ACCF), which was approximately 51.3% higher than that of the traditional titanium mesh cage.

## Introduction

Anterior cervical corpectomy and fusion (ACCF) is a common and effective treatment modality for various cervical disorders, including cervical spondylosis myelopathy, ossified posterior longitudinal ligaments, trauma, tumors, deformity corrections, infections, and rheumatoid arthritis, especially when the disease involves one or two vertebral levels^1–4^. ACCF has been widely used as a well-tolerated approach with satisfactory postoperative outcomes, owing to the direct decompression of the spinal cord and nerve root and immediate stabilization of the affected segments^5,6^. However, reconstruction after corpectomy is challenging and may be accomplished with implantation of autografts, allografts or bone substitutes^7,8^.

The use of a titanium mesh cage (TMC) with local bone grafting has become the main method for cervical reconstruction during ACCF surgery^9^. Although this method avoids the complications of the bone donor site, maintains immediate anterior column stability with good biocompatibility, and has a high fusion rate, the incidence of postoperative TMC subsidence reported in the literature is as high as 28.6–93.3%^6,10–12^. TMC subsidence may be correlated with poor clinical efficacy or poor neurological recovery. Severe subsidence will lead to symptom recurrence, deterioration of nerve function, failure of internal fixation and kyphosis of the cervical spine^13,14^. There are many risk factors related to TMC subsidence, among which patients' own conditions and operation reasons can be avoided by case screening and operation technology improvements. However, the inherent defects of TMC itself are urgent problems that need to be solved in clinical practice^12^.

At present, the contact area between the TMC and vertebral end plate is limited. The upper and lower end plates of cervical vertebrae are all irregularly curved. After TMC implantation, they are inlaid with the end plate by the dentate edge, and the adhesiveness is poor. The contact area between the TMC and end plate is small, which is point-to-face contact. Moreover, this kind of point contact causes uneven stress distribution and relatively concentrated stress, which easily causes TMC subsidence after surgery. Therefore, it is of great significance to find a new type of TMC that is in line with the anatomical structure of the cervical vertebrae of the patients, to change the point-to-face contact into the face-to-face contact, and to avoid postoperative TMC subsidence and related complications.

For this reason, an anatomic TMC system was developed, and its mechanical properties were analyzed using a three-dimensional finite element method to determine whether this novel TMC could effectively reduce the risk of subsidence after ACCF surgery and provide a reference for further biomechanical experiments.

## Methods

### Design of the novel TMC

**Figure 1:**
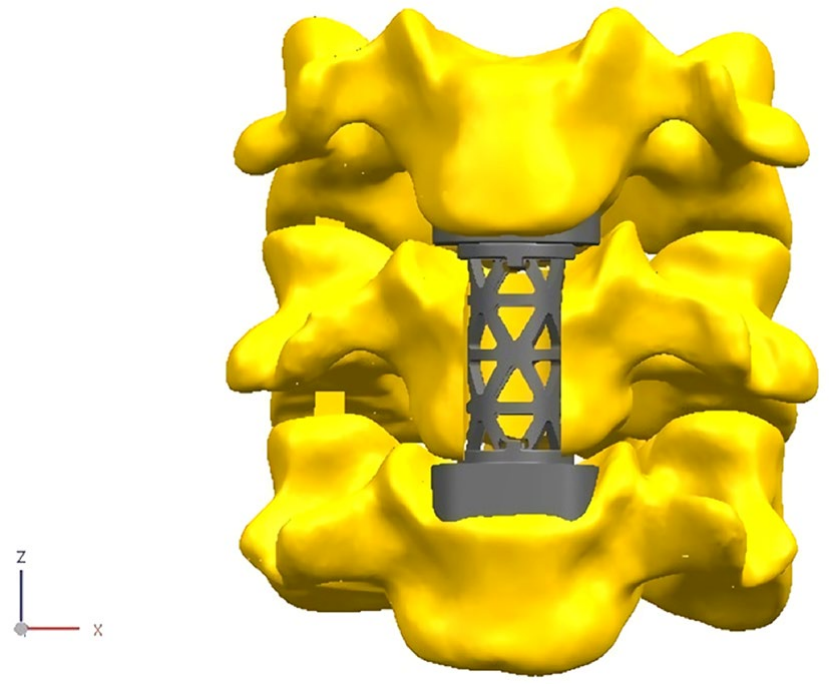
The novel anatomic titanium mesh cage for cervical spine (Solidworks 2016, SOLIDWORKS Co, USA).

The new type of anatomical TMC for the cervical spine adopts a circular hollow column structure, which is consistent with human engineering mechanics (Fig. 1). The two ends of the TMC were provided with the upper edge curved surface structure and the lower edge curved surface structure integrated with the TMC main body. In addition, the TMC main body has a hollow cylindrical structure penetrating the upper and lower curved surface structures, and a plurality of visible holes connecting the hollow cylindrical structure was arranged on the main body surface. Based on the analysis of a large number of computed tomography (CT) data of cervical vertebrae, the size of the novel TMC was classified in a step-by-step mode, and the curvature of the upper and lower edge curved surface structures matched with the anatomical data of the end plates. The upper edge curved surface structure was matched with the lower end plate of the fusion upper cervical vertebra to form face-to-face contact; the lower edge curved surface structure was matched with the upper end plate of the fusion lower cervical vertebra to form face-to-face contact.

### Subsidence testing standard

**Figure 2:**
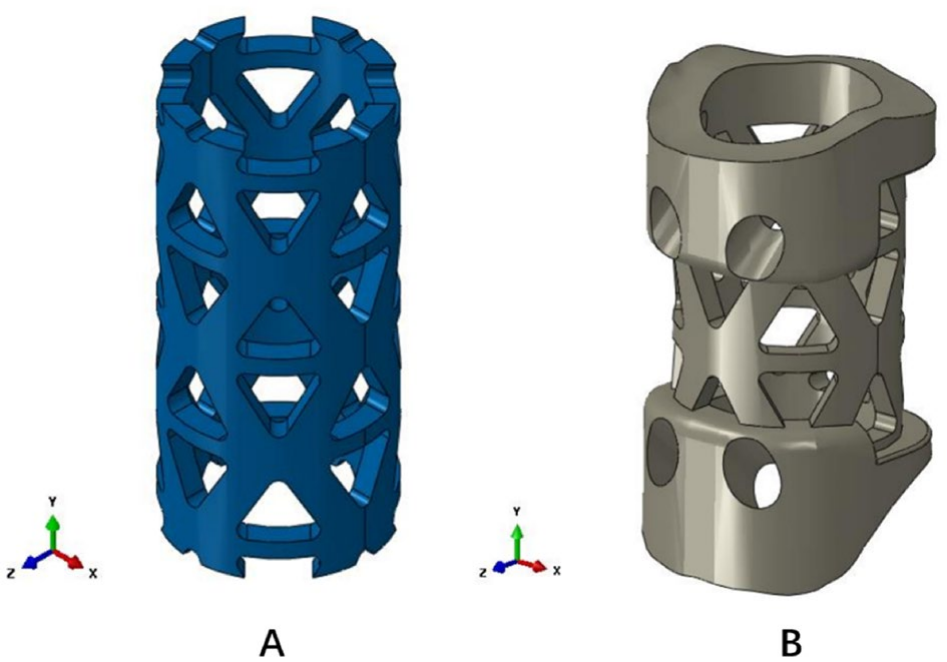
Two titanium mesh cage (TMC) models. (**A**) Traditional titanium mesh cage (TTMC); (**B**) novel titanium mesh cage (NTMC) (Solidworks 2016, SOLIDWORKS Co, USA).

In this study, three-dimensional finite element analysis was used to compare the anti-subsidence characteristics of the traditional TMC (TTMC) and novel TMC (NTMC). The two TMC models are shown in Fig. 2. The TMC, as an intervertebral body fusion device, and the subsidence testing, as described in the American Society of Testing Materials (ASTM) F2267(2011), were intended to characterize the propensity of a TCM to subside into the vertebral body end plates^15^. By modeling the subsidence testing systems as two springs in series, one can derive the relationship between the stiffness of the intervertebral body fusion device and the stiffness of the polyurethane foam blocks (simulated vertebral bodies). The relative propensity of a device to subside is quantified by a stiffness measurement, Kp (N/mm), as follows: Kp = (Ks ×  Kd)/(Kd − Ks). Kd is the stiffness of the intervertebral body fusion device, and Ks is the stiffness of the polyurethane foam blocks.

### Research model establishment and loading

The height of both TMCs was set as 25 mm, the TTMC (Medtronic Sofamor Danek, Memphis, TN) thickness was set as 1.3 mm, and the outer diameter was set as 13 mm. The thickness of the middle part of the NTMC was 1.3 mm, and the outer diameter was 13 mm. In terms of size, the two TMCs were very close. The NTMC adopted a full fit design at the interface with bone tissue. In the convergence analysis, three grid densities of 0.5, 0.7 and 1 are selected, respectively. According to the calculation results, 0.7 is selected as the grid density of the two calculation models.

According to the test process specified in the ASTM F2267 standard, first, the rigid pressure clamp was selected to load the displacement load along the vertical direction, the size was 5 mm, and the measured rigidity of this process was recorded as Kd. Then, the rigid pressure clamp was changed to grade 15 polyurethane foam blocks (intended to replicate the compression properties of trabecular bone). The loading process described above was repeated, and the measured stiffness of this process was recorded as Ks. Kp was calculated according to the above formula. A higher Kp measurement is generally expected to indicate that the device is more resistant to subsidence into a vertebral body.

**Figure 3:**
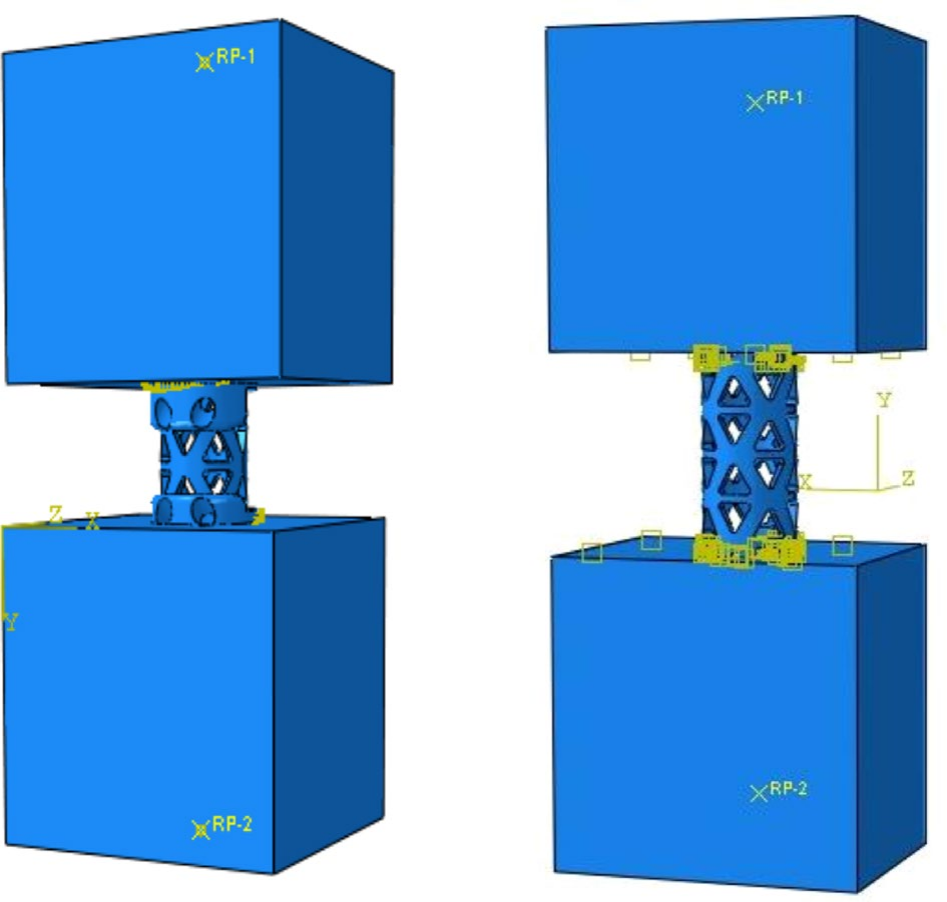
Schematic diagram of diagram of boundary conditions and load settings for the novel titanium mesh cage (NTMC) and traditional titanium mesh cage (TTMC) (Solidworks 2016, SOLIDWORKS Co, USA).

Taking the NTMC as an example, the boundary conditions and load settings were illustrated. The size of the upper and lower grade 15 polyurethane foam blocks was 40 × 40 × 60 (mm), and the upper and lower surfaces of the blocks were selected to establish coupling constraints, as shown in Fig. 3. RP1 was coupled with the upper surface of the NTMC, and RP2 was coupled with the lower surface of the NTMC. The downward displacement load of 5 mm was loaded on RP1, and the other degrees of freedom were constrained. All degrees of freedom on RP2 were constrained. The upper and lower polyurethane foam blocks and NTMC were set as face-to-face contact, the contact property was vertical hard contact, and the horizontal direction was set as "rough" to ensure that no sliding occurred in the horizontal direction during the loading process. Abaqus (2016, SIMULIA Co, USA) software was used for the mesh, and the modulus of elasticity, Poisson's ratio and other material coefficients of each part were input into the model (Table 1). Solidworks (2016, SOLIDWORKS Co, USA) software was used to establish the TTMC and NTMC models and construct the two three-dimensional finite element models of TMC subsidence testing described above.

**Table 1:** Material properties of the components of the finite element (FE) model.

Description	Young’s modulus (MPa)	Poisson’s ratio	Yield strength (MPa)
TTMC	110,000	0.3	830
NTMC	110,000	0.3	830
grade 15 blocks	123	0.3	4.9

*NTMC* novel titanium mesh cage, *TTMC* traditional titanium mesh cage.

## Results

### TTMC analysis results

**Figure 4:**
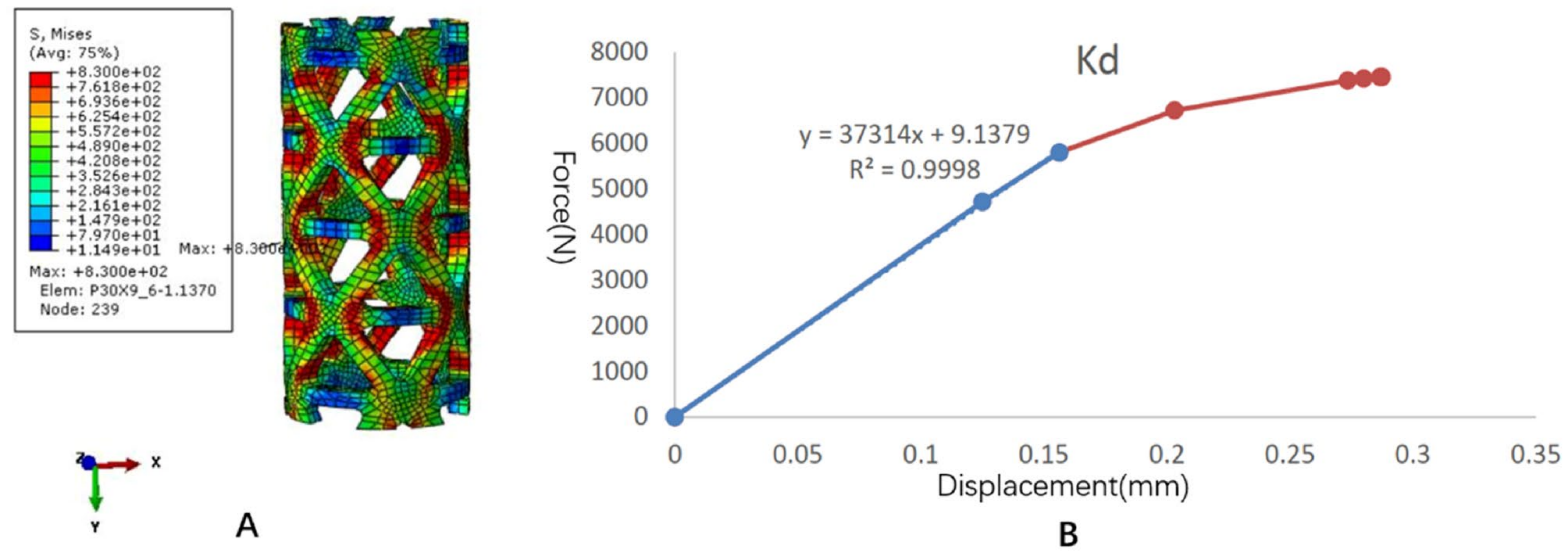
The stress distribution and the force displacement curve of the traditional titanium mesh cage (TTMC)’s Kd. (**A**) The stress distribution diagram; (**B**) the force displacement curve (Abaqus 2016, SIMULIA Co, USA).

**Figure 5:**
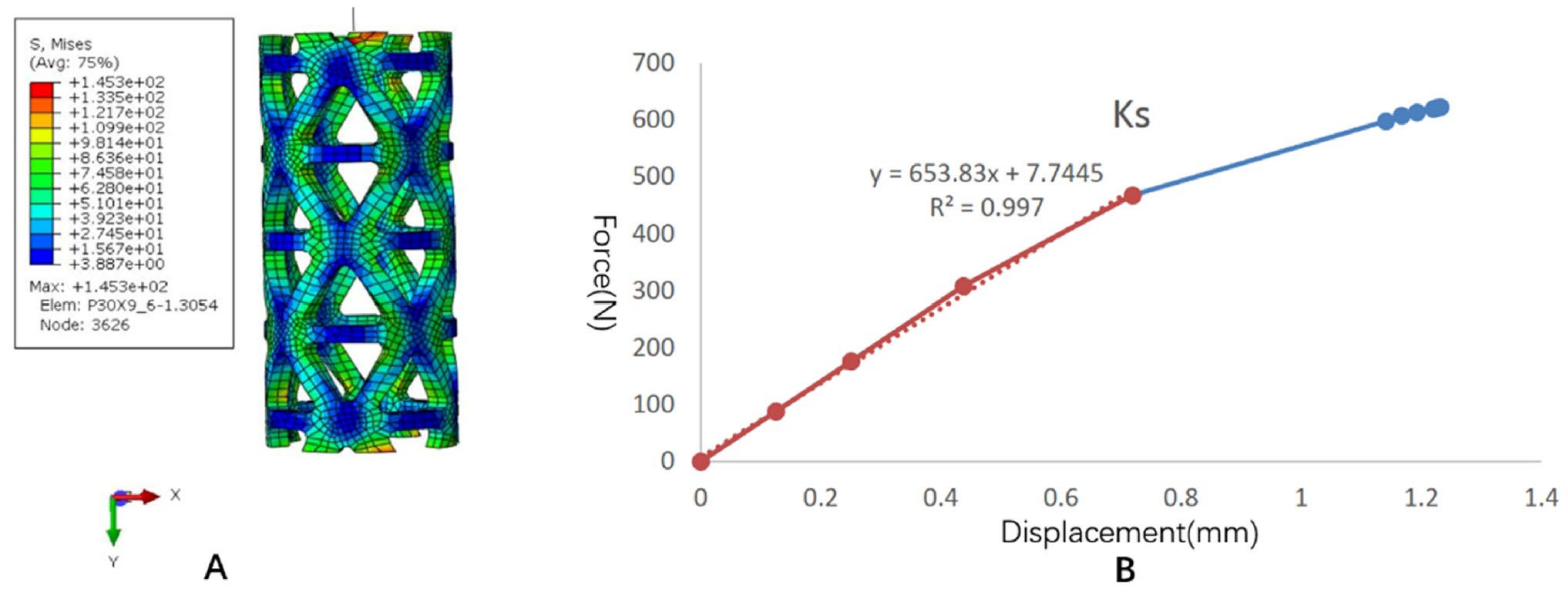
The stress distribution and the force displacement curve of the traditional titanium mesh cage (TTMC)’s Ks. (**A**) The stress distribution diagram; (**B**) the force displacement curve (Abaqus 2016, SIMULIA Co, USA).

**Figure 6:**
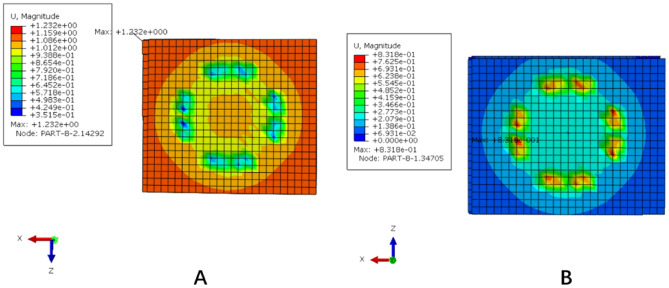
The subsidence and displacement nephogram of the traditional titanium mesh cage (TTMC). (**A**) Upper polyurethane foam blocks subsidence displacement nephogram; (**B**) lower polyurethane foam blocks subsidence displacement nephogram (Abaqus 2016, SIMULIA Co, USA).

The stress distribution and the force displacement curve of Kd are shown in Fig. 4. Although the loading was 5 mm, because the upper and lower pressure blocks were rigid bodies, the calculation stopped due to element distortion when the overall compression displacement was 0.2873 mm. However, at this time, the stiffness Kd could be obtained, and the whole TTMC yielded. After fitting the linear segment, the Kd value was 37,314 N/mm. The stress distribution and the force displacement curve of Ks are shown in Fig. 5. When the total displacement was loaded to 1.232 mm, the TTMC sank into the polyurethane foam blocks, and the Ks calculation stopped. The subsidence and displacement nephogram of the TTMC is shown in Fig. 6. At this time, the Ks value obtained was 653.83 N/mm. The Kp value of the TTMC was calculated to be 665.5 N/mm according to the formula.

### NTMC analysis results

**Figure 7:**
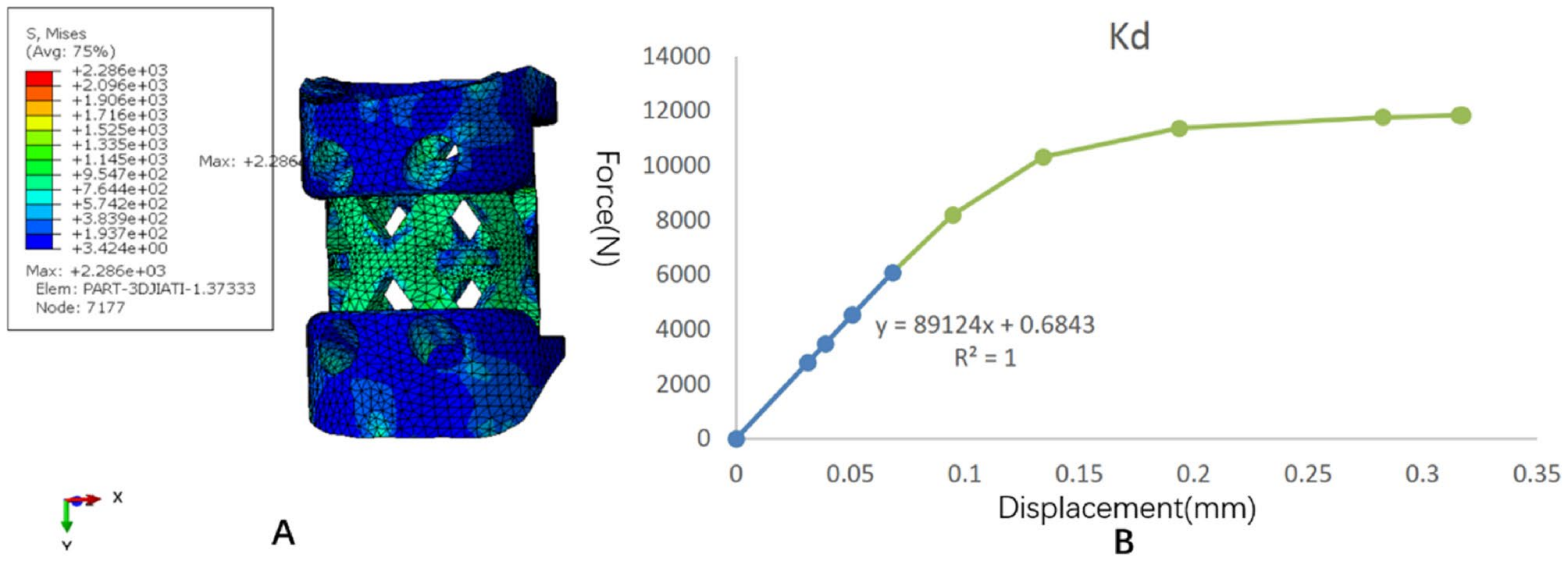
The stress distribution and the force displacement curve of the novel titanium mesh cage (NTMC)’s Kd. (**A**) The stress distribution diagram; (**B**) the force displacement curve (Abaqus 2016, SIMULIA Co, USA).

**Figure 8:**
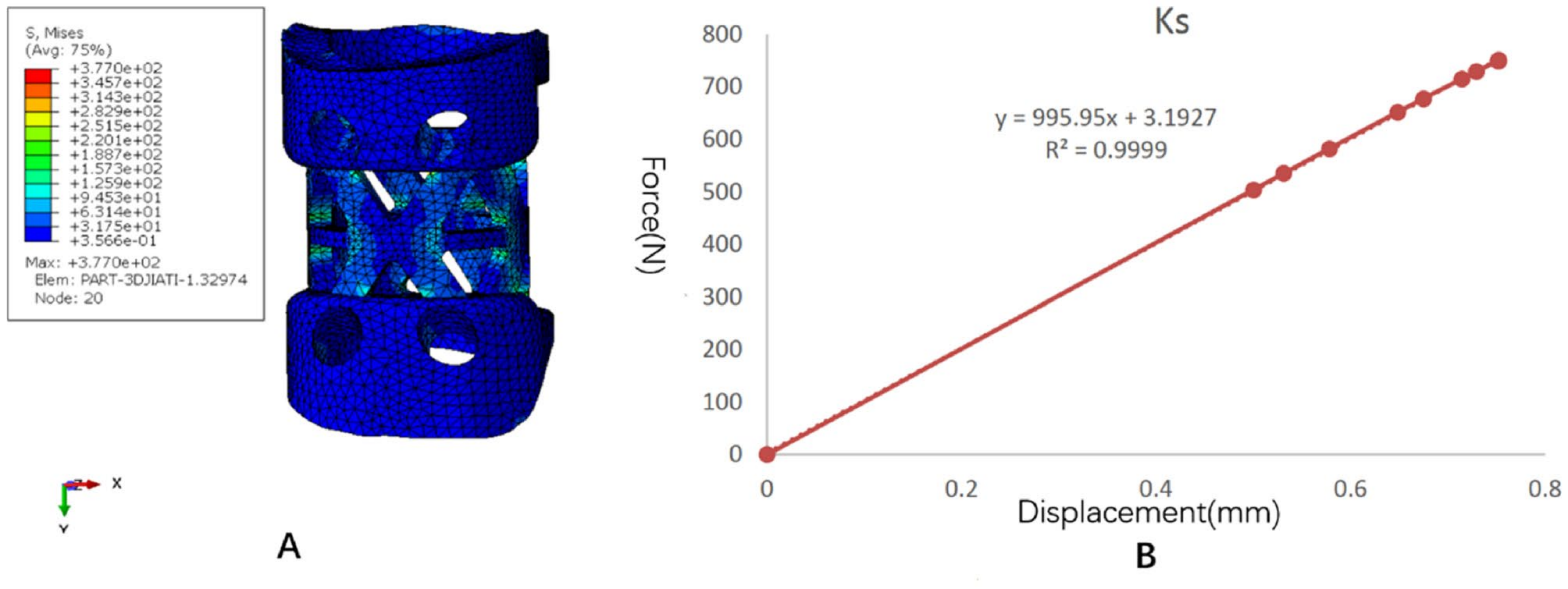
The stress distribution and the force displacement curve of the novel titanium mesh cage (NTMC)’s Ks. (**A**) The stress distribution diagram; (**B**) the force displacement curve (Abaqus 2016, SIMULIA Co, USA).

**Figure 9:**
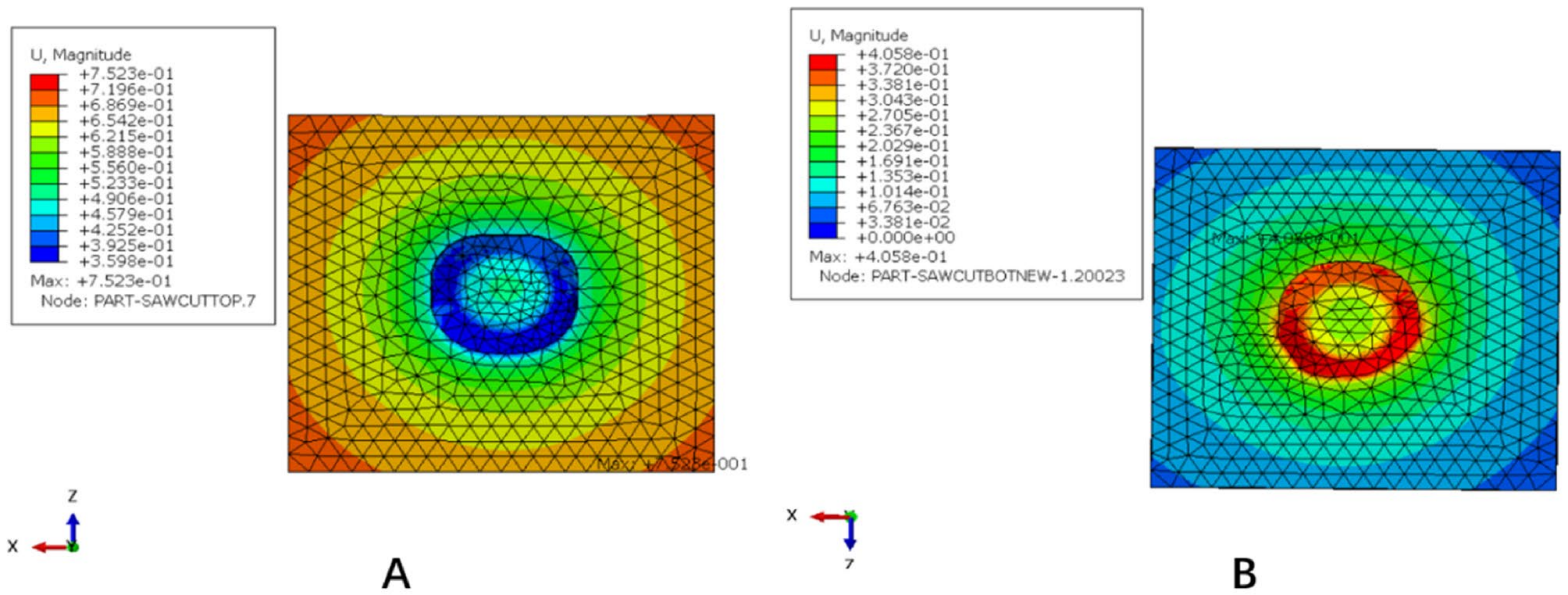
The subsidence and displacement nephogram of the novel titanium mesh cage (NTMC). (**A**) Upper polyurethane foam blocks subsidence displacement nephogram; (**B**) lower polyurethane foam blocks subsidence displacement nephogram (Abaqus 2016, SIMULIA Co, USA).

The stress distribution and the force displacement curve of Kd are shown in Fig. 7. Similar to the TTMC, although the loading was 5 mm, because the upper and lower pressure blocks were rigid bodies, the calculation stopped due to element distortion when the overall compression displacement was 0.317 mm. However, at this time, the stiffness Kd could be obtained, and the whole NTMC yielded. After fitting the linear segment, the Kd value was 89,124 N/mm. The stress distribution and the force displacement curve of Ks are shown in Fig. 8. When the total displacement was loaded to 0.75 mm, the NTMC sank into the polyurethane foam blocks, and the Ks calculation stopped. The subsidence and displacement nephogram of the NTMC is shown in Fig. 9. At this time, the Ks value obtained was 995.95 N/mm. The Kp value of the NTMC was calculated to be 1007.2 N/mm according to the formula.

### Improvement rate of anti-subsidence performance

The calculation formula of the anti-subsidence performance improvement rate is (Kp_(NTMC)_–Kp_(TTMC)_)/Kp_(TTMC)_. After calculation, the anti-subsidence performance of the NTMC was approximately 51.3% higher than that of the TTMC.

## Discussion

ACCF has long been a classic operation in anterior cervical surgery, and its curative effect is stable and reliable, but the structural integrity of the cervical spine after corpectomy remains a challenge^1,8,16^. The use of an autologous bone graft from the iliac crest is the ideal solution for the reconstruction of corpectomy defects. However, donor site morbidity has been reported in up to 25% of patients undergoing this procedure^17^, and in the cervical spine, postoperative complications such as pseudoarthrosis, graft displacement, fracture and deformity also occur occasionally^18^. Allografts and bone substitutes can avoid the morbidity associated with graft harvesting, but their use has been questioned due to the delayed union and low fusion rates^6,19,20^. Currently, different intervertebral body fusion devices have been developed to maximize anterior column stability, avoid donor-site morbidity, improve biocompatibility, and reduce instrumentation-related morbidity^6^.

Among them, the TMC has been highly praised by most surgeons with a high fusion rate^21^. Thalgott et al.^8^ reported a 100% fusion rate for multiple-level cervical corpectomy fusion using the TMC with a local bone graft. However, the TMC subsidence rate is very high and may cause instability, reconstruction failure and neurological deterioration. In a large sample size study by Chen et al.^22^ TMC subsidence was observed in 239 of 300 patients (79.7%) after ACCF surgery. In another over 8-year follow-up study by Hu et al.^23^ among the 52 patients with single-level anterior corpectomy, the probability of TMC subsidence of > 3 mm was as high as 40.4%. Through the analysis of the traditional TMC, it is clear that the main reason for subsidence is that the shape of the upper and lower ends of the TMC is not consistent with that of the end plate, and because the end surface of the TMC needs to be cut during the operation and the contact points are often sharp, it is easy to produce stress concentration and puncture the bony end plate. A TMC with sharp edges increases primary stability and resistance to early anterior displacement by cutting through the end plate, but it may also increase the chance of late subsidence^24^. This kind of point surface contact easily causes TMC subsidence.

In this study, we invented a novel type of anatomical TMC for the cervical spine. The new curved structure of the upper and lower edges of the NTMC can perfectly fit with the upper and lower end plates of the cervical spine, and it can truly achieve ideal face-to-face contact. Theoretically, the upper and lower contact surfaces designed by the NTMC according to the surface characteristics of the cervical endplate increase the contact area between the NTMC and upper and lower vertebrae. When the cervical vertebrae move, the NTMC can distribute the load onto the surface of the upper and lower vertebrae better and improve the anti-subsidence performance of the TMC.

The finite element method is an ideal tool to study the biomechanics of the spine and implanted medical devices^25,26^. This study is based on the subsidence testing standard of the intervertebral body fusion device, ASTM F2267, to analyze the anti-subsidence characteristics of the NTMC using the finite element method. According to ASTM F2267, polyurethane foam blocks model should be used for analysis, which is homogeneous, simulate the biomechanical properties of native bone^27^. In recent years, many orthopedic experiments have used polyurethane as replicate bone, and it has been confirmed that polyurethane has the same performance as human bone, and even has better consistency in structure and biomechanics^28–30^. Previous studies have shown that rigid polyurethane foams with certain densities have similar microstructure and mechanical properties compared to human cancellous bone^31–33^. As we all know, cervical body with the endplate intact has better mechanical properties than cancellous bone. However, in ACCF operation, in order to achieve better fusion rate, the endplate is often destroyed, and the TMC can directly contact with the cancellous bone. Therefore, it is reasonable to use polyurethane foams instead of cervical vertebrae after ACCF. After calculation, the Kp of the TTMC was 665.5 N/mm, while the Kp of the NTMC with face-to-face contact to the end plates was increased to 1007.2 N/mm. The Kp value is the benchmark to measure the subsidence tendency of the TMC. The larger the Kp value is, the smaller the tendency of the TMC to sink into the vertebral body. In contrast, the smaller the Kp value is, the greater the tendency of the TMC to sink into the vertebral body. Our results show that the NTMC does not easily sink compared with the TTMC, and the anti-subsidence performance of the NTMC is significantly improved by 51.3%.

Owing to the sharp footprints of the TTMC and in order to improve the contact relationship between the vertebral body and TMC, the use of end-caps has been recently suggested to increase the contact area and reduce the subsidence rate although, in theory, the TMC with end-caps also achieves face-to-face contact. However, it is essentially different from the NTMC described by us. Because the end plate is a curved structure and the TMC with end caps is a planar structure, the end cap cannot form good adhesion with the end plate. Chen et al.^22^ suggested that end caps did not sufficiently increase the contact area to resist subsidence due to the different orientations of the TMC surface and the endplates; conversely, bone fusion was delayed because of the loss of bony contact. Hur et al.^34^ conducted a comparative study on 84 patients with single-segment ACCF in the 6-year period. Subsidence was less frequent in the TMC with end-cap group (34.2%) than in the TMC without end-cap group (52.1%). However, its anti-subsidence performance was only improved by 34.4%, which is significantly less than the improvement rate of the NTMC by 51.3%. The reason may be that the curved structure of the upper and lower edges of the NTMC can effectively fit with the upper and lower end plates to provide better anti-subsidence performance.

This study has the following shortcomings as a finite element study of the anti-subsidence performance of the NTMC. First, due to the requirements of finite element technology, in the process of modeling, bone tissue and the end face of the TMC must be 100% integrated, while the TTMC has difficultly achieving such an ideal state in the actual operation process, so the actual results of the TTMC may be worse than the simulation results. Second, to facilitate the popularization and application of the NTMC, the size of the NTMC is divided into different types in a stepped mode based on the analysis of CT scan data of cervical vertebrae. In theory, the individualized 3D-printed NTMC based on the patient's cervical CT data will have better matching and anti-subsidence performance. Of course, according to the existing structure of the NTMC, a 3D printing model can also be made. In addition, in this study, only the anti-subsidence characteristics of the NTMC were defined, but its stability and effectiveness still need to be further verified.

## Conclusions

According to the predicted results of this finite element analysis, the reported novel anatomic titanium mesh cage significantly improved the anti-subsidence performance after ACCF, which was approximately 51.3% higher than the performance of the traditional titanium mesh cage.

## Data Availability

The datasets generated and/or analyzed during the current study are available from the corresponding author on reasonable request.
